# An integrated community health worker intervention in rural Nepal: a type 2 hybrid effectiveness-implementation study protocol

**DOI:** 10.1186/s13012-018-0741-x

**Published:** 2018-03-29

**Authors:** Sheela Maru, Isha Nirola, Aradhana Thapa, Poshan Thapa, Lal Kunwar, Wan-Ju Wu, Scott Halliday, David Citrin, Ryan Schwarz, Indira Basnett, Naresh KC, Khem Karki, Pushpa Chaudhari, Duncan Maru

**Affiliations:** 1 Possible, Kathmandu, Nepal; 2 Department of Obstetrics and Gynecology, Boston Medical Center, Boston, MA USA; 3 Department of Obstetrics and Gynecology, Boston University School of Medicine, Boston, MA USA; 4 Department Medicine, Division of Women’s Health, Brigham and Women’s Hospital, Boston, MA USA; 5 Harvard T.H. Chan School of Public Health, Boston, MA USA; 6 Henry M. Jackson School of International Studies, University of Washington, Seattle, WA USA; 7 Department of Anthropology, University of Washington, Seattle, WA USA; 8 Department of Global Health, University of Washington, Seattle, WA USA; 9 Department of Medicine, Division of Global Health Equity, Brigham and Women’s Hospital, 75 Francis Street, Boston, MA 02115 USA; 10 Department of Medicine, Division of General Internal Medicine, Massachusetts General Hospital, Boston, MA USA; 11 Department of Medicine, Harvard Medical School, Boston, MA USA; 12 Department of Health Services, Nepal Health Sector Programme, Ministry of Health, Kathmandu, Nepal; 13 Department of Health Services, Family Health Division, Ministry of Health, Kathmandu, Nepal; 14 Department of Community Medicine, Tribhuvan University, Institute of Medicine, Kathmandu, Nepal; 15 Department of Health Services, Ministry of Health, Kathmandu, Nepal; 16 Department of Medicine, Division of General Pediatrics, Boston Children’s Hospital, Boston, MA USA; 17 Department of Global Health and Social Medicine, Harvard Medical School, Boston, MA USA

**Keywords:** Community health workers, Reproductive health, Maternal health, Child health, Public health surveillance, Telemedicine, Implementation research, Type 2 hybrid-effectiveness-implementation, RE-AIM, Nepal

## Abstract

**Background:**

Evidence-based medicines, technologies, and protocols exist to prevent many of the annual 300,000 maternal, 2.7 million neonatal, and 9 million child deaths, but they are not being effectively implemented and utilized in rural areas. Nepal, one of South Asia’s poorest countries with over 80% of its population living in rural areas, exemplifies this challenge. Community health workers are an important cadre in low-income countries where human resources for health and health care infrastructure are limited. As local women, they are uniquely positioned to understand and successfully navigate barriers to health care access. Recent case studies of large community health worker programs have highlighted the importance of training, both initial and ongoing, and accountability through structured management, salaries, and ongoing monitoring and evaluation. A gap in the evidence regarding whether such community health worker systems can change health outcomes, as well as be sustainably adopted at scale, remains. In this study, we plan to evaluate a community health worker system delivering an evidence-based integrated reproductive, maternal, newborn, and child health intervention as it is scaled up in rural Nepal.

**Methods:**

We will conduct a type 2 hybrid effectiveness-implementation study to test both the effect of an integrated reproductive, maternal, newborn, and child health intervention and the implementation process via a professional community health worker system. The intervention integrates five evidence-based approaches: (1) home-based antenatal care and post-natal care counseling and care coordination; (2) continuous surveillance of all reproductive age women, pregnancies, and children under age 2 years via a mobile application; (3) Community-Based Integrated Management of Newborn and Childhood Illness; (4) group antenatal and postnatal care; and 5) the Balanced Counseling Strategy to post-partum contraception. We will evaluate effectiveness using a pre-post quasi-experimental design with stepped implementation and implementation using the RE-AIM framework.

**Discussion:**

This is the first hybrid effectiveness-implementation study of an integrated reproductive, maternal, newborn, and child health intervention in rural Nepal that we are aware of. As Nepal takes steps towards achieving the Sustainable Development Goals, the data from this three-year study will be useful in the detailed planning of a professionalized community health worker cadre delivering evidence-based reproductive, maternal, newborn, and child health interventions to the country’s rural population.

**Trial registration:**

ClinicalTrials.gov Identifier: NCT03371186, registered 04 December 2017, retrospectively registered.

**Electronic supplementary material:**

The online version of this article (10.1186/s13012-018-0741-x) contains supplementary material, which is available to authorized users.

## Background

Under-utilization of life saving services during the “Golden Thousand Days,” from conception to age two, continues to be a critical gap in reducing maternal, neonatal, and child morbidity and mortality in low-income countries [1–3]. Evidence-based medicines, technologies, and protocols exist to prevent many of the annual 300,000 maternal, 2.7 million neonatal, and 9 million child deaths, but they are not being effectively implemented and utilized in rural areas [4–6]. Nepal, one of South Asia’s poorest countries and over 80% rural, is a paradigmatic case of these challenges. Mortality rates are high and unequally distributed, with rural impoverished districts bearing much of the burden [7].

Community health care workers (CHWs) are an important cadre of health care workers in low-income countries where human resources for health rural health care infrastructure are limited [8–11]. As local women, they are uniquely positioned to understand and successfully navigate barriers to health care access. Since founding the Female Community Health Volunteer (FCHV) program in 1988, Nepal has relied largely on this network of lay volunteers to act as connecters between the community and the health care system. During the last three decades, the role of the FCHVs has evolved and they have taken on various activities including health promotion, distribution of preventive health commodities, and home-based health care provision, using algorithms to diagnose and treat childhood illnesses and make referrals when needed [12]. Despite the comprehensive range of services provided by FCHVs for reproductive, maternal, newborn, and child health (RMNCH), the evidence shows that under-utilization of evidence-based interventions persists [7, 13].

Policy makers and health care experts in Nepal are considering how to achieve the 2015 Sustainable Development Goals. The Ministry of Health, in addition to other researchers, has identified some major issues with the FCHV program as outlined in their 2014 national FCHV survey and report [14]. The quality of work of FCHVs varies greatly across the country, and services are not tailored to the needs of diverse populations. The various activities conducted by FCHVs are organized by several different and autonomous divisions in the central government. This leads to a lack of coordination, gaps in support and supplies, and often duplication of training and service provision [12]. As these various vertical programs have added activities, FCHVs are not participating successfully in the newer ones [14]. This may be due to the capacity of the current cadre of FCHVs to take on and integrate many new activities. The only requirement for the selection of an FCHV is that she is a married woman who lives in the community she serves. There has been no minimum education requirement, and the national survey of FCHVs in 2014 indicated that 67% of FCHVs attended school, and only 14% graduated from 10th grade (standard high school education in Nepal) [14]. Low participation may also be due to the limited time that FCHVs are spending on their work—only 3 h per day or 2 days per week. Additionally, FCHVs are very loosely integrated into the management system of the public health care system. They thus have little supervision or performance evaluation [14]. The FCHV program from the late 1980s needs to be re-envisioned to meet the Sustainable Development Goals.

Globally, there have been efforts to professionalize CHWs, including minimal education requirements and full-time employment. These efforts have been successful in increasing reproductive and neonatal health care service utilization where CHWs have appropriate training, tools, and supervision, though gaps remain where marginalized and remote populations are not being reached. Additionally, these CHW programs have not been studied at scale to understand effectiveness in larger populations, reach, and cost-effectiveness [15].

Through a public-private partnership in rural Nepal between the Ministry of Health and the healthcare delivery organization *Possible*, we built an integrated CHW intervention that employs full-time, salaried CHWs to deliver evidence-based interventions to improve maternal, newborn, and child health outcomes (see Additional file 1 for a detailed description of the intervention and Additional file 2: Figure S1 for the management structure). *Possible* has found that as rural Nepal has improved education access and retention for girls, there is a sufficient talent pool for hiring local women with a 10th grade education and higher as CHWs. Additionally, rural Nepal has rapidly become technologically connected though mobile phones and cellular data and, like many low- and middle-income countries, can take advantage of mobile applications for health.

*Possible’s* CHWs deliver community-based care facilitated by use of mobile technology [16]. CHWs monitor and promote utilization of services; provide maternal and neonatal health counseling; promote self-efficacy, social support, and emergency planning; and diagnose and refer for major causes of RMNCH morbidity. The evidence-based intervention they deliver is comprised of five integrated components: (1) home-based antenatal care (ANC) and postnatal (PNC) counseling and care coordination; (2) continuous surveillance of all reproductive age women, pregnancies, and children under age two via an integrated electronic health record system; (3) Community-Based Integrated Management of Neonatal and Childhood Illness (CB-IMNCI); (4) group ANC and PNC; and (5) the Balanced Counseling Strategy to post-partum contraception.

We have previously assessed this integrated intervention over an 18-month time-period in a geography covering a population of 33,000. The evaluation demonstrated an increase in key service utilization: institutional birth, ANC visit completion, and use of modern contraceptive methods in the postpartum period. This evaluation was conducted as a pre-post assessment, and several components of the intervention were actively being rolled out during and after the intervention period [17]. The data, however, were encouraging and call for a more rigorous evaluation at scale.

The proposed study will evaluate the efficacy and implementation process of the integrated RMNCH intervention using CHWs as a national pilot. The *Possible* team partnered with Nepal’s Ministry of Health and local health officials to refine and modify the intervention to align with the Nepal Health Sector Strategy 2015–2020 [18]. We chose a type 2 hybrid effectiveness-implementation study to evaluate the effectiveness of the integrated intervention and the implementation process [19]. We utilize the RE-AIM research framework, which has been utilized for other studies of women’s health [20–23] and child health [23, 24], to guide evaluation of the implementation process. The acronym stands for Reach, Efficacy, Adoption, Implementation, Maintenance and is designed to improve the sustainable adoption of effective, generalizable, and evidenced-based interventions that can be scaled and, ideally, shape broader policy environments.

The findings from this study will be used to inform the development of a new system for government CHWs in Nepal.

### Evidence for the integrated intervention

*Possible’s* experience in improving RMNCH in rural Nepal initially centered on facility-based expansion of services and quality improvement. A survey conducted from 2011 to 2013 showed a steep increase in utilization of birth services, with institutional birth rising from 30 to 77% [25, 26]. Logistic regression revealed that the presence of comprehensive obstetric services, belief that the hospital is the safest birth location, safety prioritization in decision-making, and higher average income all significantly contributed to this change [26]. With 23% of women still delivering outside of a facility, and minimal reach into the community, *Possible* embarked on designing a community-based strategy for RMNCH [26].

A cadre of local CHWs were employed and trained. The model was intentionally distinguished from Nepal’s longstanding FCHV program. *Possible’s* CHWs have structured management, standardized initial training, ongoing refresher training, and digitally based monitoring and evaluation which fosters accountability. They all have a minimum high school education requirement and receive a salary for full-time work. CHWs refer to government facilities including village clinics ((sub) health posts) and primary hospitals (primary health centers and district hospitals).

RMNCH priorities were defined by the Nepal government and global targets:

comprehensive antenatal, postnatal and safe delivery care, and CB-IMNCI. These interventions have all shown substantial reductions in maternal, neonatal, and early child mortality [27, 28]. CHWs visit all pregnant and postpartum women to deliver ANC and PNC counseling and prepare birth plans with women and their families. The postpartum visits include counseling for mothers alongside CB-IMNCI for their newborns and children under age two. The CB-IMNCI tool has been validated by a number of studies worldwide [29] and has widespread use globally, including in Nepal [30].

Recognizing that women were being missed and identified late in their pregnancies, *Possible* started pregnancy surveillance for all reproductive age women. Every 3 months, women are asked about their last menstrual period and offered a pregnancy test if pregnancy is suspected. Early pregnancy detection is linked to better birth outcomes, likely due to early initiation of ANC and reduction of high risk behaviors [31]. Additionally, early pregnancy awareness is important to help women with undesired pregnancies access safe and legal abortion care.

In an effort to build social support and women’s empowerment, *Possible* began group care, based upon Centering Pregnancy [32, 33], to complement home visits. This is a model of facilitated groups throughout pregnancy that include health assessments and self-care activities; education, support, and socialization; and ongoing outcome evaluation. By bringing together pregnant women of similar gestational ages, the program makes efficient use of time for antenatal counseling and testing, while allowing facilitators to focus on women’s experiences and concerns. Women also develop a network of peer support during what can be a challenging time. In the rural Nepal context, these groups became an important modality for strengthening care and increasing trust in local village clinics and decentralizing ultrasound and prenatal lab services (Bangura A et al: Measuring fidelity, feasibility, costs: an implementation evaluation of a cluster-controlled trial of group antenatal care in rural Nepal, in preparation; Thapa P et al: Group compared to individual antenatal care: a cluster-controlled trial in rural Nepal, in preparation). These life-saving diagnostic services are not generally available at government clinics and enable CHWs to identify high-risk patients who may require referral to a higher level facility for further care and delivery.

*Possible* then layered postpartum contraception onto the PNC counseling during home visits. Research on interventions to improve postpartum contraception suggests that strategies that bridge the continuum of reproductive health care are more effective than short-term, stand-alone counseling sessions [34]. *Possible* adapted a postpartum contraception counseling strategy for the community setting to bring women’s desires and concerns into a shared decision-making process. The counseling method, called the Balanced Counseling Strategy, is based on a tool developed by the Population Council that has been previously piloted in multiple countries and is in accordance with the World Health Organization’s tiered effectiveness guidelines [35]. Prior studies in clinical settings in Nepal have demonstrated increased uptake of modern contraceptive methods, especially of long-acting reversible contraception, with the use of the Balanced Counseling Strategy [35–38].

There is limited, but growing evidence that mobile technology tools can enhance CHW effectiveness in the realm of reproductive health [39–41]. *Possible’s* CHWs utilize the smartphone application CommCare (Dimagi) for surveillance, care coordination, decision support, and enhanced counseling [42]. The application allows for simultaneous data collection, which is the primary source of information for quantitative evaluation of the entire integrated intervention.

## Methods

### Study aims

We will conduct a type 2 hybrid effectiveness-implementation study of an integrated RMNCH intervention within an approximate population of 300,000 people in rural Nepal. We will study the integrated intervention as it is rolled out in a stepped fashion in two districts in rural Nepal, Achham and Dolakha.

This intervention integrates five evidence-based approaches for reproductive, maternal, newborn, and child health: (1) home-based ANC and PNC counseling and care coordination; (2) continuous surveillance of all reproductive age women, pregnancies, and children via mobile application; (3) CB-IMNCI; (4) group antenatal and postnatal care; and (5) the Balanced Counseling Strategy to post-partum contraception (described in more depth in Additional file 1 and Additional file 2: Figure S1). These integrated programmatic interventions are implemented by the non-profit organization *Possible* in partnership with the Ministry of Nepal.

### Study site

The study will take place in Achham and Dolakha districts of Nepal. The intervention will be implemented in step-wise fashion across the village clusters in coordination with district authorities and study staff.

Achham is located in the hilly far western region of Nepal, home to the highest under-five mortality rates in the country at 82 (one in 12) deaths per 1000 live births [43]. This region has suffered the consequences of war, long-standing poverty, and a concentrated HIV epidemic. *Possible* implements community health care delivery programs and independently manages the government-owned district-level Bayalpata Hospital, a 50-bed facility that serves approximately 80,000 outpatients and 2000 inpatients per year. CHW services in Achham will be expanded to 40 village clusters with approximately 200,000 people.

The second location will be in Dolakha district, one of the hardest hit districts and the epicenter of the second earthquake in 2015 [44]. Upon invitation by the Ministry of Health, *Possible* began managing Charikot Hospital in Dolakha in January 2016. Charikot Hospital is a 35+ bed facility that treats approximately 50,000 outpatients each year. The intervention will be scaled up district-wide during the course of the study. Forty village clusters with approximately 100,000 people will be included.

### Study population

The study population will include women of reproductive age (between ages 15–49) and children from 0 to 24 months of age enrolled in continuous surveillance, home-based care, and/or monthly group ANC led by *Possible’s* team in Achham and Dolakha. Specifically, this will include patients receiving health care services at Bayalpata Hospital in Achham and Charikot Hospital in Dolakha or household-level care through networks of CHWs across both districts.

Staff members involved in the program will be approached for key informant interviews (KIIs) and focus group discussions (FGDs). For all qualitative methods, we will use purposive sampling aiming to maximize heterogeneity across sex, socioeconomic position, health care issues, geographic location, age, caste-class, and other attributes. KIIs will be conducted with participants of the group ANC groups, recently delivered women, women receiving antepartum and postnatal home-based care, and reproductive age women receiving pregnancy screening. KIIs will also be conducted with team members who implemented the interventions, community leaders, and government leaders.

Participants eligible for inclusion in the study include the following: (1) married, reproductive-aged women 15–49; (2) reproductive-aged women 15–49; recently delivered in past 2 years; (3) reproductive-aged women 15–49 with active pregnancy during study period and identified by a CHW serving their village; (4) children aged 0–2 of recently delivered mothers (population 2 or 3 above); and (5) health care staff members including CHWs serving village clusters, research performance site employees involved in study design, program implementation, data collection, or data analysis processes. Study populations 1–4 must reside in either Achham or Dolakha.

Individuals meeting inclusion criteria, as stated above, will be included in the study without exclusion unless (1) individuals migrate from the study before completion of any of the integrated interventions or (2) individuals explicitly request exclusion from the study and decline to consent to the study.

### Description of the standard of care

Currently the standard of care for RMNCH at the community level in both districts of the study is the care delivered by FCHVs. FCHVs are women selected by mother’s groups in each of the villages and serve in a voluntary capacity, receiving some financial incentives but no salary. After an initial 18-day training, they receive a certificate and a medical kit including basic medications, such as iron and folate tablets for pregnancy and oral rehydration salts for diarrheal diseases. They have short training sessions when new programs are added to their workload and have minimal ongoing training. FCHVs are minimally supervised by the government village clinic staff, and they do not use mobile tools in a widespread fashion [45, 46]. They are engaged in health promotion, distribution of preventive health commodities (such as iron-folate tablets and family planning methods), and home health care provision, using CB-IMNCI algorithms to diagnose and treat childhood illnesses and make referrals when needed [12]. There may be other non-governmental programs operating in Achham and Dolakha working with local FCHVs and health care facilities. We will take note of these programs and the services they are providing to understand how our results may be affected.

### Study design

This is a type 2 hybrid effectiveness-implementation research study examining an integrated RMNCH intervention. This intervention integrates the five evidence-based approaches described above, focused on the “golden 1000 days” from conception through age two. We plan to study the impact on population health outcomes using a pre-post quasi-experimental design with stepped implementation as it is rolled out by CHWs in Achham and Dolakha. We will assess the implementation process using the RE-AIM framework, closely examining reach, efficacy, adoption, implementation, and maintenance.

We will evaluate the intervention as it is rolled out in stepped implementation. Implementation will occur in sub-populations of about 20,000 at a time (including village clusters of 1000 to 6000 people). Full implementation of the integrated intervention in any sub-population will take 6 months. Approximately 80 clusters across the two sites will be involved in the study, with a total population of approximately 300,000. This is incorporated into the power calculations below.

In this pragmatic trial, village cluster units are determined by Nepal administrative boundaries. Enrollment and timing of enrollment are based upon local government decision-making and as such cannot be randomized. Data collection is developed and integrated within the routine course of care, because this is the approach we have found to be ethical, acceptable, and affordable in our context. This has the following implications: (1) the number of villages per cluster is variable, (2) interval between new clusters being ramped up can be variable from the scheduled start times, and (3) baseline data occurs at the beginning of each step rather than at study start.

### Sample size

This implementation research study uses a census methodology rather than a representative sample. The programmatic intervention aims to reach all individuals within the catchment area population given the research performance site’s care mandate to serve this population. All individuals in the catchment area population, as defined under study site above, who meet the inclusion criteria will be approached for inclusion into the programmatic intervention, for which their de-identified clinical data will be analyzed towards the primary aim. The sample size is determined by the overall population covered in the two districts of 300,000, (rather than calculated via a sample size formula) within which we anticipate approximately 8000 deliveries per year or 12,000 during the first 18 months of the study during which enrollment will occur.

To calculate power for the primary outcome of institutional birth rate, we consider a Poisson regression model with 0.05 significance level and two-sided power of 80 or 90%. We further assume a 1-year exposure mean per child over the course of the intervention. We will consider models where the secular trend and other covariates explain 25 or 50% of the total variance in mortality. We assume intra-cluster correlation of outcomes would range from 0.2 to 0.8 for design considerations. Based on these parameters and recent advances in stepped wedge methodology [47], we calculated a design effect ranging from 0.3 to 0.8 to account for the wedge design. To be conservative, we further included a multiplicative design effect of 2.5 to account for cluster randomization, resulting in an overall design effect of 1.8 at a maximum.

### Data collection

#### Quantitative data

All data for evaluating effectiveness outcomes (Aim One) and implementation outcomes (Aim Two) will originate from *Possible’s* CommCare system. At the community level, data are entered via the CommCare mobile application. The CommCare system automatically syncs data in near-real time on secure cloud servers. In addition, CommCare allows *Possible* to audit and track changes made in any record.

All quantitative data will be collected during program implementation, and baseline/endline measurements will signify the start/endpoints of the intervention in a particular village cluster as determined by stepped-wise implementation. All data for evaluating implementation measures related to content protocol fidelity, participation, and coverage will originate either from the CHW CommCare application or from CHN observer checklists that are filled out by a supervising CHN.

#### Qualitative data

Ethnographic approaches have had some success in describing the health risks of vulnerable, global populations. We have previously developed a locally validated seven-category framework for qualitatively describing the implementation processes [48]. Data collection will be implemented in three phases. The first phase is largely exploratory, in which we develop an exhaustive list of potential categories related to the existing seven-domain framework. The aim is to validate the existing framework and to elaborate other factors that might lie outside the framework or that challenge how the framework resonates with experiences on the ground. The second phase will consist of participant observation, FGDs and free listing exercises with clinicians, health care facility management team members, and patients. In addition to KIIs and FGDs, ideals expressed in interviews and group discussions will be observed and compared to actual practice. The third phase consists of managing, organizing, and analyzing the qualitative data collected in phases one and two to develop a comprehensive list of strengths, weaknesses, and opportunities. The conclusions of these different methods will be used to help plan for future implementations of similar interventions.

All qualitative data will be stored on a Research Electronic Data Capture (REDCap) database. REDCap is a secure, web-based application designed to support data capture for research studies, providing (1) an intuitive interface for validated data entry, (2) audit trails for tracking data manipulation and export procedures, (3) automated export procedures for seamless data downloads to common statistical packages, and (4) procedures for importing data from external sources. REDCap user access will be defined so that researchers only have access to de-identified study data.

Qualitative data will be reviewed after they are aggregated on a weekly basis following data collection events. Once data are stored into REDCap, all paper copies of data form will be stored in a locked cabinet within a locked room in Bayalpata Hospital or Charikot Hospital. Once all data are fully inputted and validated for quality, all paper copies will be destroyed. REDCap data will be deleted 1 year after study end.

### Data analysis

#### Analysis for specific Aim 1: effectiveness

For assessing effectiveness, we focus on three population health outcomes as shown in Table 1. We hypothesize the integrated intervention will lead to a 15% increase in the institutional birth rate—Primary Outcome 1—among reproductive age women in the study population over the three-year study period. We hypothesize the integrated intervention will lead to a two death per 1000 reduction in the under-two mortality rate—Secondary Outcome 1—in the study population over the three-year study period. We hypothesize the integrated intervention will lead to a 10% increase in modern PPCPR—Secondary Outcome 2—among recently delivered women (defined as having had a live birth in the preceding 24 months) in the study population over the three-year study period.

**Table 1:** Evaluation framework

Aim	Outcome/RE-AIM element	Indicator	Definition
Effectiveness	Primary outcome 1: institutional birth	Institutional birth rate	% women delivering at a facility with a skilled birth attendant
	Secondary outcome 1: child mortality	Under-2 mortality rate	# child deaths before age 2 per 1000 live births
	Secondary outcome 2: post-partum contraception	Post-partum contraceptive prevalence rate	% post-partum women using a modern form of contraception
Implementation	Reach	Home visit coverage	-% children under age 2 years receiving a CHW home visit, measured monthly -% pregnant women receiving a home visit by a CHW, measured monthly -% reproductive aged women receiving a pregnancy surveillance home visit, measured quarterly -% postpartum women receiving 1+ balanced counseling session, measured biannually
		Group participation	% scheduled participants completing group sessions, measured monthly
		Session completion	% scheduled group sessions completed, measured monthly
		Demographic, geographic barriers and facilitators	Mapping households, describing barriers/facilitators to individuals’ access, and identifying contributors to variation/inequities
	Efficacy	Pregnancy identification	% all pregnancies identified in first 12 weeks
		ANC completion	-% women at 9+ months gestation completing 4+ ANC visits at a health care facility and eligible for government financial incentive out of total # of women delivered, measured monthly -% pregnant women who receive an obstetric ultrasound by their due date, measured monthly -% pregnant women with prenatal labs completed by 6 months gestation, measured monthly
		Exclusive breastfeeding prevalence	% infants age 0–5 months who are exclusively breastfed, measured monthly
		Pediatric pneumonia incidence	# new cases of pneumonia in children under age 2 years, measured monthly
		Pediatric stunting prevalence	# children under age 2 years whose length-for-height/height-for-age is < 2 SDs WHO child growth standards median, measured monthly
		Unmet contraceptive need	% eligible reproductive-age women within 12 months postpartum who desire to either stop or postpone childbearing for the next 2 years who are not currently using a modern contraceptive method or have a repeat unintended pregnancy while not using modern contraception, measured monthly
		Contraception method mix	% women within 12 months postpartum using each method of modern contraception, measured quarterly
		Contraception initiation and continuation	Contraceptive initiation and continuation probabilities at 3, 6, 12, and 24 months after method initiation, measured quarterly
	Adoption	Village cluster adoption	% intended village clusters receiving intervention
		Timely adoption	% intended village clusters rolling-out intervention within 3 months of schedule
		CHW adoption	-# CHWs hired/trained in intervention implementation -# CHWs who resign
		Government adoption	-% group care sessions co-facilitated by a government health worker -# collaborative care meetings with government partners during implementation
	Implementation	Supervision model	-% scheduled supervision field visits completed, stratified by CHN and district -# data review meetings held with CHWs and CHNs, measured quarterly
		Group care content fidelity	% planned topics covered at each session, measured monthly
		Home visit fidelity	-% women with “adequate” number of home visits received -% appropriate ANC topics covered during each pregnancy care home visit -% child health topics covered during each under 2 home visit -% balanced counseling points covered at each postpartum contraception counseling session, measured biannually
		Referral completeness	% referrals completed as prescribed by the clinical algorithm
		Implementation challenges	Exploratory and hypothesis-generating as revealed through FGDs and KIIs
	Maintenance	Total intervention cost	Cost of each intervention component and total costs using the Time Defined Activity Based Costing (TDABC) method
		Cost per unit	-Intervention cost per 1000 people -Projected cost to scale intervention nationally
		Maintenance cost	% breakdown of maintenance costs (personnel, materials, and other)
		Facility vs. community costs	% of costs of health care divided between facility level and community level
		Geographic cost variation	Variation of costs between village clusters and districts
		Out-of-pocket patient costs	% costs of health care divided between facility level and community level
		Integrated intervention cost-effectiveness	Pre/post intervention marginal effectiveness for primary outcomes, not account for secondary effects (e.g., increasing the costs of care through increased cesarean sections)

The primary predictor is the effect of the intervention measured in 3-month intervals throughout the study period, comparing to the FCHV standard-of-care control at baseline. Calendar month will be included as a separate covariate to control for the overall secular trend. Variables will be considered as both nominal and continuous (linear effect) predictors, and the generalized linear model framework will be used to estimate effect of time-varying repeated measure intervention implementation over the several steps of the wedged design. Differential impact from time of intervention will be evaluated with test of month × intervention interaction. Models will be fit using generalized estimating equations, e.g., using SAS Proc Genmod, to calculate valid standard errors in the presence of repeated measures over time and possibly correlated outcomes at the village cluster level. Assumptions of over- or under-dispersion will be examined closely, and an estimated scale parameter or negative binomial models will be used as needed.

#### Analysis for specific Aim 2: RE-AIM implementation analysis

Here, we use the RE-AIM framework to evaluate the implementation process (see Table 1). (R)each in a remote area is vital to success. We will assess coverage of and participation in the integrated intervention for each reproductive age woman, pregnant and postpartum woman, and under-two child. Additionally, we will analyze barriers and facilitators to coverage and participation.

For (E)fficacy, we look at our integrated intervention’s ability to successfully identify pregnancies in the first trimester, pediatric cases of illness, and women with an unmet need for contraception. If these are successfully identified and appropriate care is delivered, then we will see an impact on the outcomes in Aim One. Additionally, we look at secondary outcomes not included as the main measures of effectiveness in Aim One: exclusive breastfeeding, modern contraception initiation and continuation, and government ANC visits.

Community level (A)doption is largely dependent upon the local political and governance context. Process measures for community-level adoption include the number of village clusters that roll out the intervention, the time to roll out relative to the stated target date, and the number of CHWs hired and trained. We will also assess commitment to the intervention and its various components through semi-structured KIIs with managers and CHWs.

We will assess (I)mplementation fidelity to the planned interventions. Using a qualitative approach, we will explore implementation challenges from a variety of perspectives, including key stakeholders, government partners, NGO staff, and community members. We will assess the quality of the intervention from the communities’ and patients’ perspective through in-depth KIIs.

We will assess the all-inclusive costs of the interventions for (M)aintenance. The time-defined activity based costing method will be deployed for measuring costs at the beneficiary level by clearly identifying activities, personnel capacity required, materials, and equipment usage, as well as other general and administrative costs required. We will assess costs incurred for each intervention type, for varying levels of pregnancy care, and variability of costs across village clusters and districts. The cost measurement method will help in identifying cost optimizing methods without reducing effectiveness of the interventions to ensure judicious use of resources. The analysis will inform policy decisions that will lead to affordable and scalable interventions. This method will not fully capture all the economic benefits due to the intervention. Evidences suggest strong benefit-cost ratio for early investments in health with improved economic outcomes due to higher human capital, increased productivity and cognitive ability, and demographic dividends.

For each of the quantitative outcomes in RE-AIM, we will apply a similar generalized estimating equations methodology using 3-month intervals from data routinely collected by the mobile tool. For the qualitative component of the implementation analysis that includes FGDs, data analysis will be ongoing and iterative. Each step of exploratory interviewing and observation will shape the questions in the subsequent steps. The analysis of free list data will concentrate on two measures of an item’s salience. The first is the frequency with which any informant mentions each term, and the second is the salience index as computed by ANTHROPAC [49]. These measures will be used to estimate the salience of individual categories and to define the boundaries of the domain into which they fall. An additional measure of interest is the correlation between each informant’s responses and the group as a whole. Interviews and discussions will be partially transcribed and coded according to the Grounded Theory Method [50], a rigorous procedure for identifying themes in text and for developing theoretical models of the relationships among themes [51].

For the cost-benefit analysis, we are interested in the marginal, direct costs of the intervention rather than potential secondary cost implications (e.g., cost savings by decreased outpatient visits or cost increases by increased cesarean sections). We will assess the costs of RMNCH delivery using a simplified variant of time-driven activity-based costing method where time is allocated by clinician interview rather than by observation. At present, the main national health care policy questions in Nepal involve how to gain better access to essential services guaranteed by the Constitution and per policy but which are incompletely implemented in practice. Policymakers are most interested in the marginal, direct costs of community-based care provision rather than the larger effects that might result.

## Discussion

At the conclusion of this three-year study, we hope to assess the effectiveness and implementation of a complex and integrated RMNCH intervention delivered by CHWs in two rural districts of rural Nepal, covering a population of 300,000. As Nepal takes steps towards achieving the Sustainable Development Goals, these data will be useful in the detailed planning of a professionalized CHW cadre reaching the country’s rural population. Designed as a national pilot, the policy potential of this study is clear. However, there is likely international utility as well, given Nepal’s challenges are not unique. As a systematic review of CHW programs globally concluded, there is a need among policy makers in low- and middle-income countries for detailed implementation data, as well as data from programs brought to scale [15].

In addition to the content of the evaluation, the methodology used to conduct the evaluation will be an important contribution to the literature of implementation science. We have chosen to combine two important approaches, the type 2 hybrid effectiveness-implementation approach and the RE-AIM framework. The RE-AIM framework serves to break the implementation evaluation into five separate components, providing focus on the most important aspects of implementation for sustainable adoption of evidenced-based interventions.

The effectiveness evaluation is pragmatic and avoids the ethical challenges with having a control group when testing evidenced-based interventions. Additionally, evaluating “stepped” implementation over a three-year period allows the data to incorporate and account for some temporal trends. Each step is measured immediately prior to implementation, which is likely a stronger approach than historical controls. Perhaps the most important aspect of the chosen evaluation is that it is built within a developing health care system, and conducting research within this system strengthens data systems and quality for non-research purposes.

There are limitations, however, to this “quasi-experimental” approach. When there is no concurrent control group, causal inferences are difficult to make. Lack of randomization can lead to both measured and unmeasured biases. When examining implementation, our study will likely be affected by the baseline variation that exists in the large geography we are covering. We will measure and account for distances to health care facilities, socio-economic variation, and health care personnel quality. We cannot, however, account for other programs on RMNCH running in the same catchment area populations. We will assess for what programs are being run and at what intensity; however, we cannot control for them. Additionally, the standard of care provided by government FCHVs may look slightly different across the catchment area populations; however, the baselines should account for these differences.

Despite the limitations, this study holds promise for providing high-quality data and detailed implementation information on a complex and integrated RMNCH intervention in a resources-limited and rural setting. We hope to contribute to the literature on the implementation and effectiveness of CHWs for the very important work of addressing the health of women and children in Nepal and similar settings.

## Additional files

Additional file 1: Detailed description of the intervention (DOCX 16 kb)

Additional file 2: **Figure S1.** Supervision structure (PNG 50 kb)

## Abbreviations

ANC: Antenatal care
CB-IMNCI: Community-Based Integrated Management of Newborn and Childhood Illness
CHN: Community health nurse
CHW: Community health care worker
FCHV: Female Community Health Volunteer
FGD: Focus group discussion
KII: Key-informant interview
PNC: Postnatal care
RMNCH: Reproductive, maternal, newborn, and child health
