# Supplementary material for: An integrated community health worker intervention in rural Nepal: a type 2 hybrid effectiveness-implementation study protocol
Source: Implement Sci. 2018 Mar 29;13:53. doi: 10.1186/s13012-018-0741-x (PMC5875011; doi:10.1186/s13012-018-0741-x)
Supplement: Supplementary file 1 — Detailed description of the intervention (DOCX 16 kb) [file 13012_2018_741_MOESM1_ESM.docx]

An integrated community health worker intervention in rural Nepal: A type 2 hybrid effectiveness-implementation study protocol

**Supplemental File 1: Detailed description of the intervention**

Five intervention components:

1. Home-based ANC and PNC counseling and care coordination: Once a pregnant woman is identified, CHWs carry out ANC home visits once a month at the pregnant woman’s home. These visits include ANC counseling specific to gestational age, screening, and referral for high-risk pregnancies. CHWs discuss all referrals and high-risk pregnancies with their supervisors, to ensure appropriate follow-up and to help trouble-shoot issues with access to care. The CHWs work with all pregnant women and their families on a birth plan, discussing and planning for transportation, social support, and finances at the time of birth. After delivery, a woman is visited in her home and postnatal counseling (PNC) commences. During monthly visits through the first year postpartum, she is screened for danger signs and counseled on breastfeeding, postpartum depression, and contraception (described in more detail below).
2. Continuous surveillance of all reproductive age women and children under age two: Continuous surveillance is the backbone for all data collection and the primary system to both identify and provide care for patients. When initially moving into a new area of coverage the first phase is for the CHWs to visit every household, use a GPS mobile application to geotag the house, assign a household ID number, collect demographic household information, and register all married women of reproductive age and all children under age two into the continuous surveillance data system. Once enrollment is complete, CHWs visit households with eligible participants every three months and record vital events, as well as note migrated individuals. Households with newly eligible participants are enrolled into the care delivery interventions as the CHW becomes aware of them. If a married woman of reproductive age is suspected of pregnancy according to a screening algorithm, she is then offered a point-of-care urine pregnancy test. For pregnant women, postpartum women, and children under age two, surveillance increases to monthly visits, and care is delivered, as described below.
3. Community-Based Integrated Management of Newborn and Childhood Illness: A CHW carries out home visits once a month once a child under age two has been identified, either during enrollment or ongoing surveillance. CHWs provide CB-IMNCI counseling specific to the child’s age and, using a symptom and clinical signs-based algorithm, the CHWs screen and provide referrals for diarrhea, malnutrition, malaria, respiratory illness, and measles. CHWs discuss all referrals with their supervisors, to ensure appropriate follow-up and to help trouble-shoot issues with access to care.
4. Group antenatal and postnatal care: CHWs and government nurse midwives jointly conduct group visits at rural village clinics. Women from the surrounding villages present on a specified day for their 4^th^ and 6^th^ month check-ups and on another day for their 8^th^ and 9^th^ month check-ups. During the earlier gestational age group, women receive a check-up, antenatal labs, and counseling on 1^st^ and 2^nd^ trimester issues. During the later gestational age group, women receive a check-up, obstetric ultrasound, and counseling on 3^rd^ trimester and postnatal issues. In both groups, women participate in discussion and facilitated peer-to-peer problem solving around barriers to care.
5. The Balanced Counseling Strategy to post-partum contraception: During the 8^th^ month antenatal visit, the CHWs review available contraception methods. Following delivery, at months one, five, and ten, CHWs engage women in a more in-depth counseling module. The structured counseling modules are adapted from the Balanced Counseling Strategy, an interactive contraceptive counseling method. The balanced counseling algorithm is accompanied by visuals that the CHWs can use to explore a woman’s reproductive intentions and guide their counseling. CHWs focus the counseling on methods that are most relevant to each woman’s reproductive goals and preferences. In addition to balanced counseling, CHWs provide referrals and help to facilitate women accessing their chosen method of contraception. CHWs also provide information on birth spacing, condom use for sexually transmitted disease prevention, emergency contraception use, and reinforce the criteria for the lactational amenorrhea method.

Mobile Care Coordination System:

All CHWs are provided with an Android mobile phone equipped with CommCare, an open-source application developed by Dimagi that can be customized to support frontline workers in low-resource communities. CommCare includes capabilities for data collection, decision support, task reminders, and counseling tools. The mobile application can be accessed and used in the field offline and the data are uploaded at a later time when online services are accessible. All CHWs are provided with an internet device that can be used to upload data from their own homes. The data collected can then be synthesized and exported to Excel spreadsheets and statistical software for cleaning and analysis.

CHW Supervision Structure:

To ensure accountability, the CHW program has a rigorous management system that includes regular in-person monitoring of CHWs and quality assurance (see Staffing Structure below). Community Health Nurses (CHNs), who are trained clinicians and registered with Nepal Medical College, are the CHWs’ direct supervisors. CHWs are monitored by these direct managers, once weekly and in-person, while CHWs are seeing individuals at their homes. This system guarantees regular on-the-job coaching of CHWs to improve adherence to clinical protocols as well as patient education and counseling. Additionally, CHWs attend twice-monthly meetings at a central office location. These meetings include a group-training component, where skills are refreshed and common concerns can be shared and discussed. During the twice-monthly meetings, CHWs also have individual meetings with their direct supervisors. During these 1:1 meetings, they review patient care data on high-risk pregnancies and referrals, and performance data and outcome indicators. The CommCare application tracks patient-specific and CHW-specific metrics, enabling the CHNs to take a data-driven approach in targeted training and quality improvement initiatives to particular geographies and particular CHWs, ensuring ongoing optimization for patient care. Program Associates manage CHWs and CHNs covering a population of approximately 40-50,000 depending on geographic and administrative boundaries. The Program Associate’s role is focused on training, ensuring outcome indicators are being met, and management support for the CHNs. There is one District Manager who leads the entire Community Health care program as shown in Supplemental Figure 1.
