# Supplementary figures and images for: An integrated community health worker intervention in rural Nepal: a type 2 hybrid effectiveness-implementation study protocol
Source: Implement Sci. 2018 Mar 29;13:53. doi: 10.1186/s13012-018-0741-x (PMC5875011; doi:10.1186/s13012-018-0741-x)

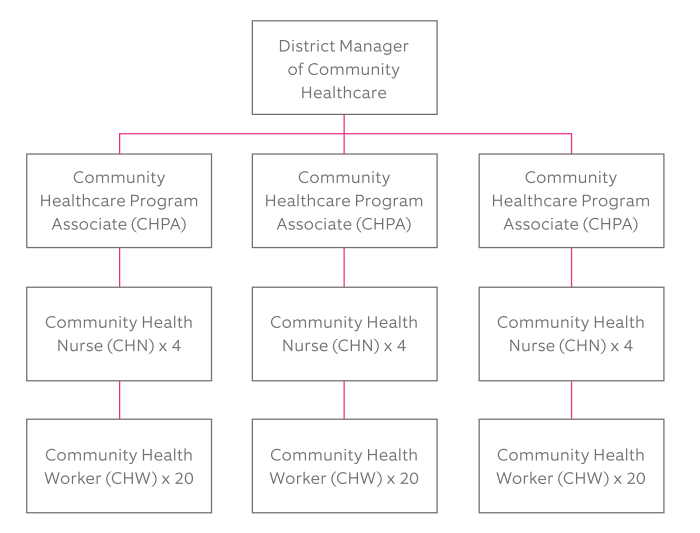

Supplement: Supplementary file 2 — Figure S1. Supervision structure (PNG 50 kb) [file 13012_2018_741_MOESM2_ESM.png]
